# The O-Antigen Flippase Wzk Can Substitute for MurJ in Peptidoglycan Synthesis in *Helicobacter pylori* and *Escherichia coli*

**DOI:** 10.1371/journal.pone.0161587

**Published:** 2016-08-18

**Authors:** Wael Elhenawy, Rebecca M. Davis, Jutta Fero, Nina R. Salama, Mario F. Felman, Natividad Ruiz

**Affiliations:** 1 Department of Biological Sciences, University of Alberta, Edmonton T6G 2E9, Alberta, Canada; 2 Department of Microbiology, The Ohio State University, Columbus, OH 43210, United States of America; 3 Division of Human Biology, Fred Hutchinson Cancer Research Center, Seattle, WA 98109, United States of America; 4 Department of Molecular Microbiology, Washington University School of Medicine, St. Louis, MO 63110, United States of America; Centre National de la Recherche Scientifique, Aix-Marseille Université, FRANCE

## Abstract

The peptidoglycan (PG) cell wall is an essential component of the cell envelope of most bacteria. Biogenesis of PG involves a lipid-linked disaccharide-pentapeptide intermediate called lipid II, which must be translocated across the cytoplasmic membrane after it is synthesized in the inner leaflet of this bilayer. Accordingly, it has been demonstrated that MurJ, the proposed lipid II flippase in *Escherichia coli*, is required for PG biogenesis, and thereby viability. In contrast, MurJ is not essential in *Bacillus subtilis* because this bacterium produces AmJ, an unrelated protein that is functionally redundant with MurJ. In this study, we investigated why MurJ is not essential in the prominent gastric pathogen, *Helicobacter pylori*. We found that in this bacterium, Wzk, the ABC (ATP-binding cassette) transporter that flips the lipid-linked O- or Lewis- antigen precursors across the inner membrane, is redundant with MurJ for cell viability. Heterologous expression of *wzk* in *E*. *coli* also suppresses the lethality caused by the loss of *murJ*. Furthermore, we show that this cross-species complementation is abolished when Wzk is inactivated by mutations that target a domain predicted to be required for ATPase activity. Our results suggest that Wzk can flip lipid II, implying that Wzk is the flippase with the most relaxed specificity for lipid-linked saccharides ever identified.

## Introduction

The cell envelope of most bacteria contains a cell wall composed of peptidoglycan (PG) [1]. Bacteria build the PG matrix around their cytoplasmic membrane by polymerizing a disaccharide-pentapeptide into glycan chains that are crosslinked by peptide bonds [2, 3]. The resulting PG polymer protects cells from osmotic lysis in hypotonic environments, confers cell shape, and serves as an anchor to which envelope structures can be attached. Given these important roles in bacterial physiology, it is not surprising that inhibiting PG biogenesis is lethal under most conditions and that many antibiotics function by inhibiting this process [4].

**Fig 1:**
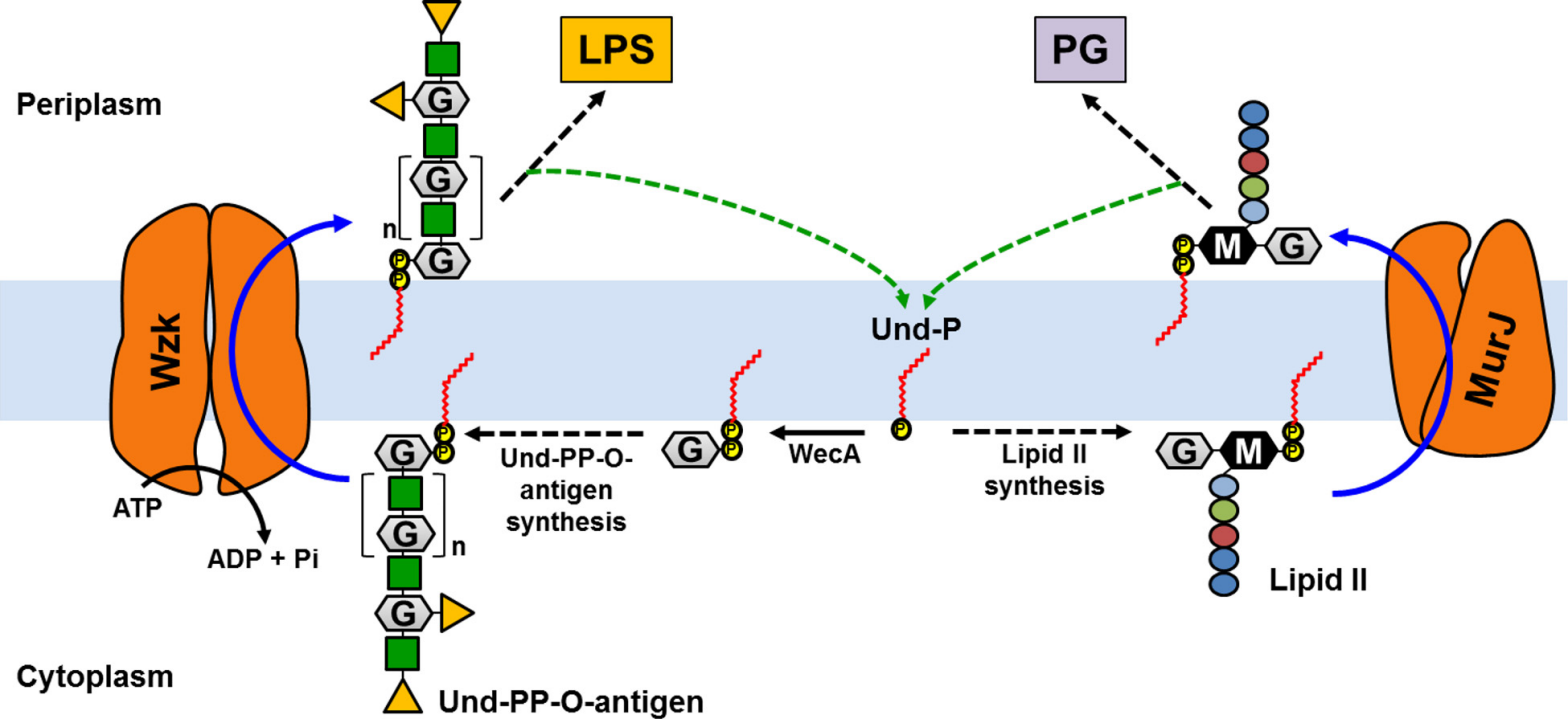
MurJ and Wzk translocate Und-PP linked saccharides across the cytoplasmic membrane. Nucleotide-linked sugars (not shown) are used to build the PG precursor lipid II and the O-antigen precursor Und-PP-O-antigen on the membrane-bound lipid carrier Und-P. The first Und-P glycosyltransferase in each respective pathway are MraY (not shown) and WecA. After each Und-PP-linked intermediate is synthesized in the inner leaflet of the cytoplasmic membrane, flippases MurJ and Wzk translocate them across the bilayer. At the periplasmic leaflet of the cytoplasmic membrane, the disaccharide-pentapeptide component of lipid II is used to build glycan chains that are crosslinked into the preexisting PG matrix, while the O polysaccharide portion of Und-PP-O-antigen is transferred onto LPS molecules by the WaaL ligase (not shown). After these steps, the lipid carrier is recycled (green dotted arrows). The O polysaccharide is composed of *N*-acetyl glucosamine (GlcNAc, grey hexagon labeled G), galactose (green squares), and fucose (orange triangles); the disaccharide in lipid II is composed of *N*-acetyl muramic acid (MurNAc, black hexagon labeled M) and GlcNAc (grey hexagon labeled G), while the pentapeptide is composed of L-Ala (light blue circle), D-Glu (green circle), *meso*-2,6-diaminopimelic acid (*meso*-A_2_pm, red circle) and two D-Ala (dark blue circle) residues.

The PG biogenesis pathway is highly conserved among PG-producers, although there can be some differences in the composition of the disaccharide-pentapeptide and in the complexity, thickness and modifications of the final PG structure [3, 5, 6]. In Gram-negative bacteria, *Escherichia coli* has been the model organism for most studies on PG biogenesis [7]. Early studies determined that in *E*. *coli*, the disaccharide-pentapeptide is composed of GlcNAc-MurNAc-L-Ala-γ-D-Glu-*meso*-A_2_pm-D-Ala-D-Ala, which is synthesized at the inner leaflet of the cytoplasmic (or inner) membrane (Fig 1) using cytoplasmic nucleotide-linked sugars and the lipid carrier undecaprenyl pyrophosphate (Und-PP) [8–12]. The resulting lipid-linked PG precursor, known as lipid II, must then be translocated across the membrane so that it can be used by the transglycosylases that build the PG glycan chains in the periplasm (Fig 1) [7, 13]. Clearly, translocation (or flipping) of lipid II across the cytoplasmic membrane is an obligatory step in PG biogenesis; however, lipid II flipping remains poorly understood and the identification of lipid II flippases has been controversial [14, 15]. In fact, three proteins, MurJ, AmJ, and FtsW, have been proposed to be lipid II flippases but only MurJ and AmJ have been shown to be required for the translocation of lipid II in cells [16–20].

MurJ (formerly MviN) was initially proposed to be the lipid II flippase in *E*. *coli* mainly because 1) it is essential for PG biogenesis and, therefore, viability, 2) its depletion leads to the accumulation of lipid-linked and nucleotide PG precursors, and 3) it belongs to the MOP (multidrug/oligo-saccharidyl-lipid/polysaccharide) exporter superfamily of proteins, which includes flippases of substrates similar to lipid II [16, 17, 21]. Additional structure-function analyses demonstrated that the first 12 of the 14 transmembrane domains of MurJ adopt a structure similar to that of multidrug transporters of the MOP exporter superfamily [22]. Specifically, these 12 transmembrane segments organize into two six-helix bundles that fold into a V-shaped structure with a solvent-exposed central cavity that contains several charged residues that are essential for function [22, 23]. More recently, a chemical genetics approach to inactivate MurJ and an *in vivo* lipid II flippase assay were developed and combined to demonstrate that rapid inactivation of MurJ with a small molecule results in the accumulation of lipid II in the inner leaflet of the cytoplasmic membrane of *E*. *coli* cells [20]. Thus, MurJ is required for lipid II translocation across the cytoplasmic membrane of *E*. *coli* and has structural features that are essential for its function and similar to those found in other transporters. Consequently, the simplest explanation of this collection of data is that MurJ is the lipid II flippase in *E*. *coli* [20].

Putative *murJ* orthologs are present in PG producers and their essentiality has been demonstrated in several species of both Gram-negative and Gram-positive bacteria [16, 17, 24–27]. Cross-complementation between distant homologs has also been reported. For example, heterologous expression of the *murJ* ortholog from *Streptococcus pyogenes* in *E*. *coli* can complement the loss of the native *E*.*coli murJ* (*murJ*_*EC*_) even though MurJ_EC_ and MurJ_SP_ only share 22% of amino acid sequence identity [23, 28]. Unexpectedly, in *B*. *subtilis*, the MurJ homolog (MurJ_BS_, formerly YtgP) is not essential, although it can functionally substitute in *E*. *coli* for MurJ_EC_ [29, 30]. This paradox was recently explained by demonstrating that *B*. *subtilis* possesses AmJ (formerly YdaH), a protein that is functionally redundant with MurJ_BS_ that can also substitute for MurJ_EC_ in *E*. *coli* [18]. Accordingly, *B*. *subtilis* single mutants lacking either MurJ_BS_ or AmJ are viable but a mutant lacking both proteins is not [18].

Transcription of *amJ* is under the control of σ^M^, a sigma factor that is induced by cell wall stress [18]. This type of regulation favors the idea that AmJ plays a direct role in PG biogenesis under certain conditions. However, AmJ is predicted to have six transmembrane domains and is neither widely conserved among bacteria nor related to MurJ [18]. This raises the question of whether AmJ is solely a dedicated lipid II flippase or a promiscuous flippase that can translocate various substrates including lipid II. This question is especially relevant because, as described below, bacteria synthesize a variety of envelope glycoconjugates whose biosynthetic pathways include lipid II-like intermediates, some of which are translocated by flippases with relaxed substrate specificity [31, 32].

Bacteria synthesize many oligo- or poly- saccharides linked to the lipid carrier Und-PP in the cytoplasmic leaflet of their cytoplasmic membrane. Like lipid II, these lipid-linked precursors must be flipped across the membrane to be used in the synthesis of envelope components such as capsules, O antigens, wall teichoic acids, and glycoconjugates used in *N*-glycosylation [33, 34]. Several types of membrane transporters implicated in flipping Und-PP-linked intermediates have been described: 1) those belonging to the MOP exporter superfamily such as Wzx proteins and MurJ [21, 33, 35]; 2) ATP-binding cassette (ABC) transporters [36]; 3) synthase-dependent transporters [33]; and 4) unrelated transporters such as AmJ and one composed of ArnE and ArnF [18, 37]. How these transporters recognize their respective Und-PP-linked intermediate is poorly understood, although it is clear that some are capable of flipping more than their cognate substrate [31, 32].

An example of a translocase with relaxed substrate specificity is Wzk, the ABC transporter involved in lipopolysaccharide (LPS) synthesis in the Gram-negative bacterium *Helicobacter pylori* [32]. In this bacterium, Wzk is required for the synthesis of O antigens, which are made of repeats of Lewis X or Lewis Y antigens. These structures mimic human glycans playing a role in interactions with the host and are regulated by phase variation [38, 39]. Wzk is homologous to PglK, the ABC transporter that translocates the Und-PP-heptasaccharide that *Campylobacter jejuni* uses in *N*-glycosylation of proteins [31, 40]. It was shown that when produced in an *E*. *coli* strain that expresses the *N*-glycosylation system from *C*. *jejuni*, *H*. *pylori* Wzk (Wzk_HP_) could substitute for PglK [32]. Moreover, Wzk_HP_ was also able to flip the Und-PP-O16 antigen precursor that *E*. *coli* uses to modify its LPS. Thus, Wzk can flip a variety of Und-PP-oligo- and poly- saccharides [32].

Interestingly, in *H*. *pylori* MurJ (MurJ_HP_) is not essential although transposon disruption of *murJ*_*HP*_ confers defects in cell shape characteristic of a faulty PG biogenesis pathway [41]. In this study, we demonstrate that MurJ_HP_ becomes essential in the absence of Wzk, suggesting that both MurJ_HP_ and Wzk translocate lipid II in *H*. *pylori*. Furthermore, we show that Wzk can substitute for MurJ_EC_ in *E*. *coli*, and that this complementation requires an active ATPase domain. Collectively, these results suggest that *H*. *pylori* Wzk has the most relaxed specificity for lipid-linked saccharides ever characterized as it promotes the delivery of complex O-antigen, protein glycosylation and peptidoglycan precursors across the inner membrane.

## Materials and Methods

### Growth conditions and reagents

*H*. *pylori* were grown in Brucella broth with 10% fetal bovine serum (BB10) or on Columbia agar plates containing 10% horse blood (HB) in a microaerophilic environment (10% O_2_, 10% CO_2_, 80% N_2_) as described [41]. For resistance marker selection HB agar plates were supplemented with 15 μg/mL chloramphenicol or 25 μg/mL kanamycin. For *E*. *coli* cultures, lysogeny broth (LB) and glucose M63 minimal broth were prepared as described previously [42]. Solid media were prepared by adding 15 g/l of agar. Except for recombineering [43], all liquid cultures were grown under aeration at 37°C and their growth was monitored by optical density at 600 nm (OD_600_). When appropriate, kanamycin (25 μg/ml), ampicillin (125 μg/ml), 5-bromo-4-chloro-indolyl-β-D-galactopyranoside (X-gal, 20 μg/ml), and isopropyl β-D-1-thiogalactopyranoside (IPTG) were added.

### *H*. *pylori* strain construction

Strains are listed in S1 Table. Targeted disruption of *wzx* was accomplished using PCR SOEing [44] with primers 1153_for_out, 1153#2a, 1153Kan#3, 1153Kan#4 (S2 Table) leading to replacement of 399 bp of the coding sequence with *aphA3* conferring resistance to kanamycin [45]. The resulting PCR product was used directly for natural transformation of LSH100. Genomic DNA was prepared from kanamycin resistant clones by Wizard genomic DNA purification kit (Promega) and the correction integration event was confirmed by PCR analysis with primers 1153_for_out and 1153#2a [46]. A mutation in *wecA* was recovered by sequencing random clones from a transposon mutant library created in strain G27 [47]. Genomic DNA prepared from a clone with a transposon insertion at genomic position 1644261 (position 651 of the 1002 bp gene) was used for natural transformation of LSH100 followed by selection on chloramphenicol containing plates. Primes1516_rev_in and 1519_rev_in (S2 Table) were used to confirm the integration event by PCR on genomic DNA prepared from selected clones.

### Generation of Wzk variants

Site-directed mutagenesis was employed to generate inactive variants of *Helicobacter pylori* J99 Wzk. Primer pairs WzkS405A fw and rv, WzkD524A fw and rv, WzkE525A fw and rv (S2 Table), were used in separate PCR reactions to amplify pIH23 (pEXT20 encoding *wzk*) [32]. The methylated template DNA was selectively digested with DpnI (New England Biolabs), while the amplified plasmid variants were used to transform *E*. *coli* DH5α (S1 Table). The resulting clones were used to purify the plasmids expressing *wzk* variants. All point mutations were confirmed by sequencing. To test the expression of Wzk and its variants in *E*. *coli* DH5α, different strains were grown to mid-logarithmic phase. Expression of *wzk* alleles was induced with 0.5 mM IPTG for 4 hrs. OD_600_-normalized cell lysates were separated on 12% SDS-polyacrylamide gel electrophoresis (PAGE), followed by immunoblotting with anti-histidine polyclonal antibody (Santa Cruz Biotechnology, Inc.). The membrane was incubated with IRDye conjugated anti-rabbit antibody to visualize the bands using Odyssey infrared imaging system (LI-COR Biosciences).

### *In vivo* glycosylation assay of Wzk activity

The ability of *H*. *pylori* Wzk to flip UndPP-linked glycans was tested in *E*. *coli* strain SCM6, which contains mutations in the oligosaccharide translocase and O-antigen ligase [48]. We should note that because the *E*. *coli* strains we used are K-12 strains, they do not produce O antigen [49]. *E*. *coli* SCM6 carrying pACYC*pglK*mut (encoding the *C*. *jejuni* glycosylation machinery with a mutation in the translocase gene *pglK* [50] and pIH18 (encoding histidine-tagged AcrA as the acceptor protein) was transformed with either pIH23 (encoding Wzk) [32] or its variant pIH23E525A (encoding WzkE525A). As a negative control, the empty vector pEXT20 [51] was transformed instead of pIH23. To monitor Wzk *in vivo* activity, AcrA glycosylation in different *E*. *coli* SCM6 strains was detected via immunoblotting. Cell lysates, obtained from OD_600_-normalized overnight cultures, were separated on 12% SDS-PAGE. Following separation, proteins were transferred to a nitrocellulose membrane and were probed using mouse anti-histidine monoclonal antibody and rabbit anti-*C*. *jejuni* glycan polyclonal antibody. The membrane was incubated with IRDye conjugated anti-mouse and anti-rabbit antibodies to visualize the bands using Odyssey infrared imaging system (LI-COR Biosciences, Lincoln, NE).

### Construction of *E*. *coli murJ* complementation reporter strain

Previously, we developed a strain that reports on the ability of mutant *murJ* alleles to complement a chromosomal Δ*murJ* allele [22]. Here, we modified this system to test the ability of the plasmids carrying the different *H*. *pylori wzk* alleles described above to complement the Δ*murJ* allele in *E*. *coli*. The *bla* gene that confers resistance to ampicillin in pRC7MurJ [22] was replaced with a *kan* cassette that confers resistance to kanamycin using recombineering as follows. Primers BlaP1 and BlaP1 (S2 Table) were used to amplify the *kan* cassette from pKD4 [52]. The resulting PCR product was introduced into recombineering strain DY378 [43] carrying pRC7MurJ and kanamycin-resistant recombinants were selected at 30°C on LB containing kanamycin. Replacement of resistance markers was confirmed and the resulting plasmid named pRC7KanMurJ.

The *murJ* complementation reporter strain was derived from the wild-type strain MG1655 [53] and constructed as follows. First, the Δ*proC*::*kan* allele from the Keio collection [54] was introduced into MG1655 by P1 transduction. The resulting kanamycin-resistant NR2865 strain was used in another P1 transduction using TB28 (*proC*^*+*^ Δ*lacIYZA*::*FRT*) as donor and selecting for growth on glucose M63 minimal plates lacking proline. Because *proC* and *lacZ* can be co-transduced, *lacZ* transductants were identified as white colonies on glucose M63 minimal plates containing IPTG and X-gal. The resulting MG1655 *proC*^*+*^ Δ*lacIYZA*::*FRT* strain was named NR2869. The Δ*pyrC*::*kan* allele from the Keio collection [54] was then introduced into NR2869 by selecting for kanamycin resistance. The *kan* gene was excised in the resulting transductant using the FLP-recombinase produced by pCP20 [55]. Then pRC7KanMurJ was introduced into resulting strain to generate NR2874 [MG1655 Δl*acIZYA*::*FRT* Δ*pyrC*::*FRT* (pRC7KanMurJ)]. NR2874, a pyrimidine auxotroph that cannot grow in minimal medium because of the Δ*pyrC*::*FRT* allele, was then crossed via P1 transduction with *pyrC*^+^ Δ*murJ*::*kan* donor NR1648 [22] and *pyrC*^*+*^ transductants were selected on glucose M63 minimal plates containing IPTG and X-gal. When the Δ*murJ*::*kan* was acquired by co-transduction with *pyrC*^*+*^, the desired MG1655 Δ*lacIZYA*::*FRT* Δ*murJ*::*kan* (pRC7KanMurJ) transductants appeared as stably blue colonies that could not lose pRC7KanMurJ. One such transductant was named NR2890. We discarded transductants that formed white (or sectored blue/white) colonies because they retained the wild-type *murJ* allele and therefore lost pRC7KanMurJ.

### Complementation of *E*. *coli murJ* mutant with *H*. *pylori wzk*

Plasmids pEXT20, pIH23, and pIH23 derivatives encoding ATPase-deficient Wzk variants WzkS405A, WzkD524A, and WzkE525A were introduced into strain NR2890 [MG1655 Δ*lacIZYA*::*FRT* Δ*murJ*::*kan* (pRC7KanMurJ)] and transformants were selected on LB solid medium containing ampicillin and X-gal to yield merodiploid strains NR2919, NR2920, NR3649, NR3648, and NR3650, respectively. Purified transformants were then plated onto LB solid medium supplemented with ampicillin, X-gal, and various concentrations of IPTG to induce expression of *wzk* alleles. Only NR2920 [MG1655 Δ*lacIZYA*::*FRT* Δ*murJ*::*kan* (pRC7KanMurJ, pIH23)] yielded a haploid strain, NR3647 [MG1655 Δ*lacIZYA*::*FRT* Δ*murJ*::*kan* (pIH23)], in the presence of 40 μM IPTG. For growth curves, strain NR3647 was grown overnight at 37°C in LB broth supplemented with 40 μM IPTG in a culture roller drum (New Brunswick Scientific). The culture was then diluted to OD_600_ 0. 1 in LB either lacking or containing 40 μM IPTG and growth was monitored by measuring OD_600_.

### Microscopy

*E*. *coli* cells were spotted (2 μl of culture) on an LB agar pad and visualized on a Nikon Eclipse Ti-E by phase contrast using a 100X oil objective lens.

## Results and Discussion

### Δ*murJ and* Δ*wzk* are synthetic lethal in *H*. *pylori*

MurJ is essential for the viability of several bacteria including *E*. *coli*, *Burkholderia cenocepacia*, *Burkholderia pseudomallei*, *Sinorhizobium meliloti*, *Staphylococcus aureus* and *Streptococcus pneumoniae* [16, 17, 24–27]. However, it is not essential in *B*. *subtilis* [29, 30] because this bacterium encodes an unrelated protein, AmJ, which can also flip lipid II [18]. Likewise, MurJ is not essential in *H*. *pylori* but its loss leads to cell shape defects [41]. To identify the factor(s) that might be redundant with MurJ in *H*. *pylori*, we first searched for AmJ homologs in the proteome of this bacterium. A BLAST search [56] did not uncover any homologs of AmJ (NCBI reference sequence NP_388304) encoded in the genomes of *H*. *pylori* strains J99 (NCBI reference sequence NC_000921) and G27 (NCBI reference sequence NC_000915.1), suggesting that a novel protein might be capable of flipping lipid II in this bacterium.

We hypothesized that Wzk might be the protein that is redundant with MurJ in *H*. *pylori* because it is an ABC transporter that can flip various Und-PP-linked oligo- and poly- saccharides across the cytoplasmic membrane [32]. Since lipid II translocation is required for viability, we reasoned that if indeed MurJ and Wzk are redundant in *H*. *pylori*, null *murJ* and *wzk* alleles will be synthetic lethal. Therefore, to test for such synthetic genetic interactions, we set out to construct *murJ* and *wzk* single and double mutants in *H*. *pylori* strain G27 where their respective chromosomal alleles had been replaced with antibiotic-resistance cassettes, generating Δ*murJ*::*cat* and Δ*wzk*::*aphA3* alleles.

As previously reported, the single *murJ* and *wzk* single mutants (TSH1 and NSH203, respectively) were viable [32, 41]. We noted that even though we easily generated the *wzk*::*aphA3* single NSH203 mutant, it grew slowly. We suspected that this growth defect might be caused by the inhibition of the O-antigen biosynthetic pathway at the step where the Und-PP-linked O antigen is translocated across the membrane. Inhibiting this step would likely cause a drop in the pool of Und-P that is normally recycled after translocation of the Und-PP-linked O antigen precursor and that is required for the synthesis of the essential lipid II (Fig 1). This situation and phenotype would resemble those recently reported for *E*. *coli* strains in which pathways that utilize Und-PP-linked oligosaccharide precursors have been inhibited at steps that prevent recycling of Und-P [57, 58]. In *E*. *coli*, these defects were suppressed with mutations that prevent the irreversible synthesis of the Und-PP-linked precursor by blocking the pathway upstream. Therefore, we tested whether the growth defects we observed in the *H*. *pylori wzk*::*aphA3* single mutant NSH203 could be reversed by inactivating *wecA*, which encodes the UDP-GlcNAc:Und-P GlcNAc-1-phosphate transferase that catalyzes the first step required for the biosynthesis of the O antigen on its Und-P lipid carrier [32]. We found that in NSH208, a Δ*wecA* allele suppressed the growth phenotypes conferred by *wzk*::*aphA3*. These results suggest that like in *E*. *coli*, accumulation of dead-end Und-PP-linked intermediates causes growth defects by depleting Und-P [57, 58].

Having confirmed that neither *murJ* nor *wzk* are essential for the viability of *H*. *pylori*, we next attempted to construct a Δ*murJ*::*cat* Δ*wzk*::*aphA3* double mutant by selecting for kanamycin-resistant recombinants in the Δ*murJ*::*cat* strain TSH1 following transformation with genomic DNA obtained from the Δ*wzk*::*aphA3* strain NSH203. We failed to generate the Δ*murJ*::*cat* Δ*wzk*::*aphA3* double mutant. In contrast, we easily isolated kanamycin-resistant recombinants when the recipient was strain TSH3, a Δ*murJ*::*cat* that carries a wild-type *murJ* allele at the *rdxA* locus. These results indicate that *murJ* and *wzk* are a synthetic lethal pair.

While PG biogenesis is essential for the viability of *H*. *pylori*, biosynthesis of the O antigen is dispensable [32]. Furthermore, loss of Wzk disrupts O antigen biogenesis, indicating that MurJ cannot substitute for Wzk in *H*. *pylori* [32]. Therefore, the synthetic lethality conferred by the *murJ* and *wzk* alleles is likely the result of the loss of lipid II translocation. Consequently, we propose that in *H*. *pylori*, both MurJ and Wzk flip lipid II across the cytoplasmic membrane.

### Wzk can substitute for MurJ in *E*. *coli*

The aforementioned results, combined with previous results showing that the O-antigen flippase Wzk has relaxed substrate specificity [32], suggest that Wzk itself can flip lipid II in *H*. *pylori*. Since MurJ is required for lipid II translocation in *E*. *coli* [20], we determined whether heterologous expression of *wzk* in *E*. *coli* could complement a Δ*murJ* chromosomal allele.

We previously developed a method to easily determine whether a mutant allele of *murJ* complements a Δ*murJ* chromosomal allele and, if so, generate haploid *murJ* mutants in *E*. *coli* [22]. We adapted this method to determine if *H*. *pylori wzk* can substitute for MurJ in *E*. *coli* as follows. This Δ*murJ* complementation system employs a strain (NR2890) that carries a Δ*murJ* chromosomal allele and a pRC7-derived mini-F plasmid [59] encoding a wild-type *murJ* allele and *lacZ*. This plasmid, pRC7KanMurJ, is also deficient in properly segregating upon cell division [59]. As a result, Δ*murJ* (pRC7KanMurJ) strains frequently generate Δ*murJ* progeny that has lost pRC7KanMurJ. Because MurJ is essential, the resulting Δ*murJ* cells undergo MurJ depletion and eventually lyse [16, 17]. Therefore, viable progeny of Δ*murJ* (pRC7KanMurJ) strain NR2890 must always carry pRC7KanMurJ and thereby be LacZ^+^. However, if a complementing *murJ* allele is introduced into Δ*murJ* (pRC7KanMurJ) cells, the resulting progeny will be a mixed population containing both merodiploid cells that still carry pRC7KanMurJ (Lac^+^) and haploid cells that have lost pRC7KanMurJ (LacZ^-^). These haploid cells could be easily identified as they will produce white colonies on plates containing the LacZ indicator X-gal [22].

**Fig 2:**
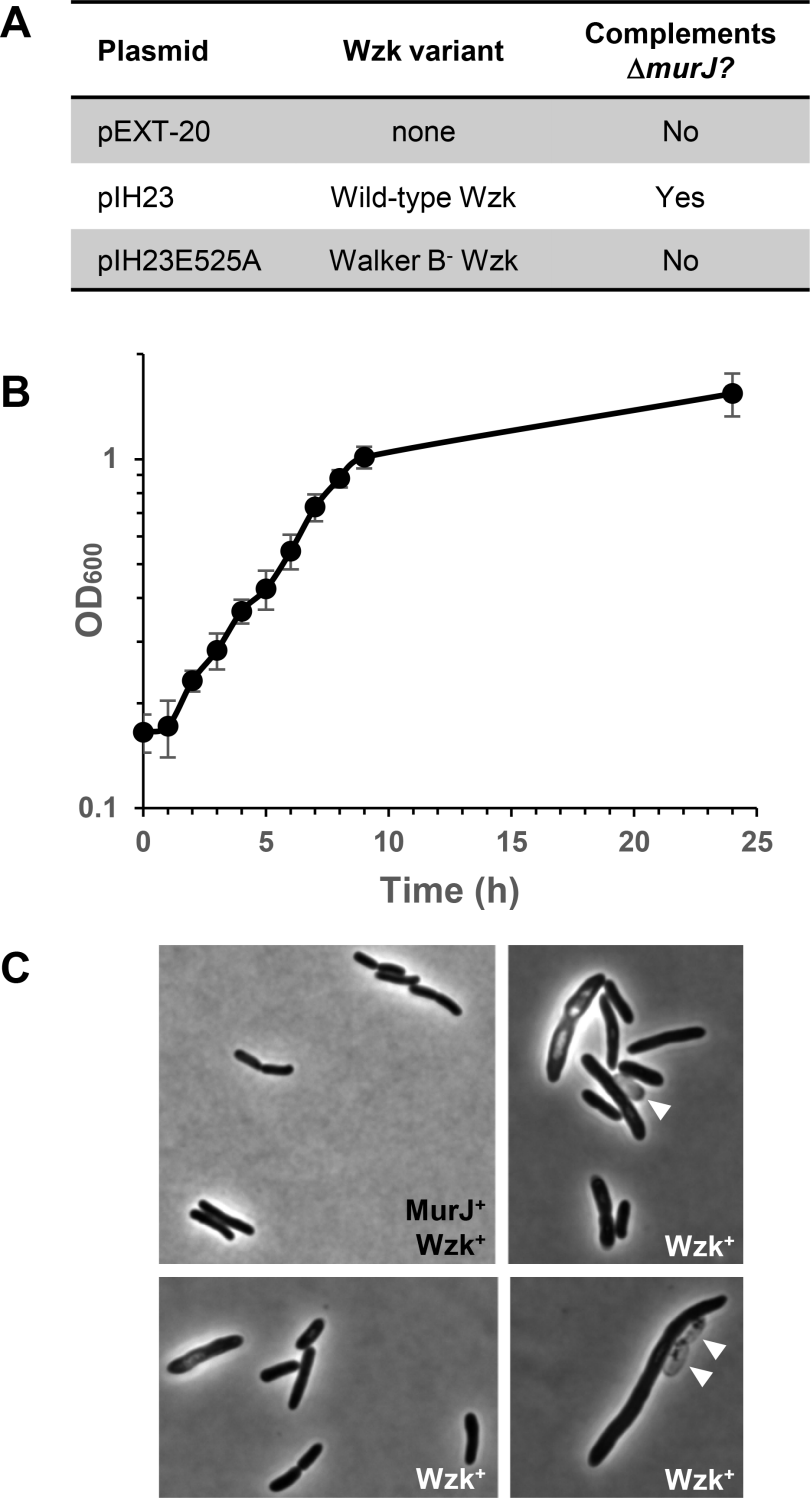
Wzk can substitute for MurJ in *E*. *coli*. (A) Summary of complementation experiments. The loss of *murJ* in *E*. *coli* can only be accomplished with a plasmid encoding wild-type *wzk*. The *wzkE525A* allele, which carries a mutation in the Walker B motif that is required for ATPase activity, cannot complement a Δ*murJ* allele. (B) Growth of *E*. *coli* strain NR3647 [MG1655 Δ*lacIZYA*::*FRT* Δ*murJ*::*kan* (pIH23)] in LB broth supplemented with 40 μM IPTG as determined by OD_600_. The data represents the average and standard deviation of six independent cultures. (C) *E*. *coli* strain NR2920 (MG1655 Δ*lacIYZA*::*FRT* Δ*murJ*::*kan* (pRC7KanMurJ, pIH23) expressing both *murJ* and *wzk* exhibits the rod-shape cellular morphology typical of wild-type *E*. *coli* cells (MurJ^+^ Wzk^+^ panel) under a 100X phase-contrast objective. Δ*murJ* cells complemented with *wzk* (Wzk^+^ panels) exhibit morphology characteristic of cells with PG defects: larger size, aberrant morphology, and lysis (marked with white arrow heads).

To determine whether *H*. *pylori wzk* can substitute for MurJ, we introduced plasmid pEXT20 and its *wzk*-encoding derivative, pIH23 [32], into Δ*murJ* (pRC7KanMurJ) cells. The respective NR2919 and NR2920 transformants only yielded blue colonies in the presence of X-gal. We then tested whether induction of *wzk* expression with IPTG could lead to complementation in NR2920. We observed that pIH23 could not complement the loss of *murJ* on plates containing ≤ 20 μM IPTG. In addition, we observed that NR2920 grew poorly on plates containing ≥ 0.2 mM IPTG likely because of the toxicity caused by overexpression of *wzk*. However, when IPTG was present at 40 μM, the Δ*murJ* (pRC7KanMurJ, pIH23) strain NR2920 readily yielded white colonies, indicating that they lost pRC7KanMurJ. Further tests demonstrated that the growth of these LacZ^-^ cells (strain NR3647) was IPTG-dependent on solid medium, indicating that their survival relies on *wzk* expression from pIH23. In contrast, the Δ*murJ* (pRC7KanMurJ, pEXT20) strain NR2919 only yielded blue colonies both in the presence and absence of IPTG. Together, these results indicate that *wzk* can complement the loss of *murJ* in *E*. *coli* (Fig 2A).

Although Wzk can substitute for MurJ in *E*. *coli*, complementation is not perfect. On LB solid media containing 40 μM IPTG, the Δ*murJ* (pIH23) strain NR3647 forms smaller colonies than its Δ*murJ* (pRC7KanMurJ) progenitor, strain NR2920, and the wild-type strain MG1655. In liquid media, the Δ*murJ* (pIH23) strain only reaches a cell density OD_600_ of ~0.4–0.6 when shaken vigorously (data not shown) and of ~1.5 when grown in a cell culture roller drum (Fig 2B), while MurJ^+^ strains can typically reach OD_600_ >3. Light microscopy also suggests that Δ*murJ* (pIH23) cells have PG defects because they exhibit shape defects and frequently undergo lysis (Fig 2C). When strain NR3647 was subjected to Wzk depletion in liquid medium lacking IPTG, we observed severe growth defects and an increase in lysis but depletion of Wzk was not complete under conditions tested (S1 Fig).

Together, these results demonstrate that production of Wzk in *E*. *coli* is sufficient to suppress the lethality caused by the loss of MurJ but not to completely restore normal PG biogenesis. Similarly, *H*. *pylori* mutants lacking *murJ* survive because they express *wzk*, albeit they exhibit cell shape defects [41]. From these results we suggest that Wzk translocates lipid II across the cytoplasmic membrane in both *H*. *pylori* and *E*. *coli* because of its relaxed substrate specificity of Und-PP-oligosaccharides. If so, the PG defects observed in MurJ^-^ Wzk^+^ cells suggest that Wzk cannot cope with the high demand for lipid II translocation in growing cells possibly because of inefficient recognition of lipid II.

### ATPase activity is required for Wzk function and its ability to substitute for MurJ in *E*. *coli*

We next tested if the ability of Wzk to substitute for MurJ is indeed dependent on its flippase activity. To do this, we constructed a non-functional Wzk variant and tested its ability to complement the loss of *murJ* in *E*. *coli*. The Und-PP-O-antigen flippase Wzk belongs to the ABC transporter superfamily of proteins [32]. ABC transporters hydrolyze ATP and utilize the energy released from ATP hydrolysis in the cytoplasm to undergo conformational changes that result in the transport of substrates across membranes [60]. Since Wzk is predicted to rely on ATP hydrolysis to function as a flippase, we constructed non-functional variants of Wzk by changing residues that are highly conserved and essential for the ATPase activity of ABC transporters.

**Fig 3:**
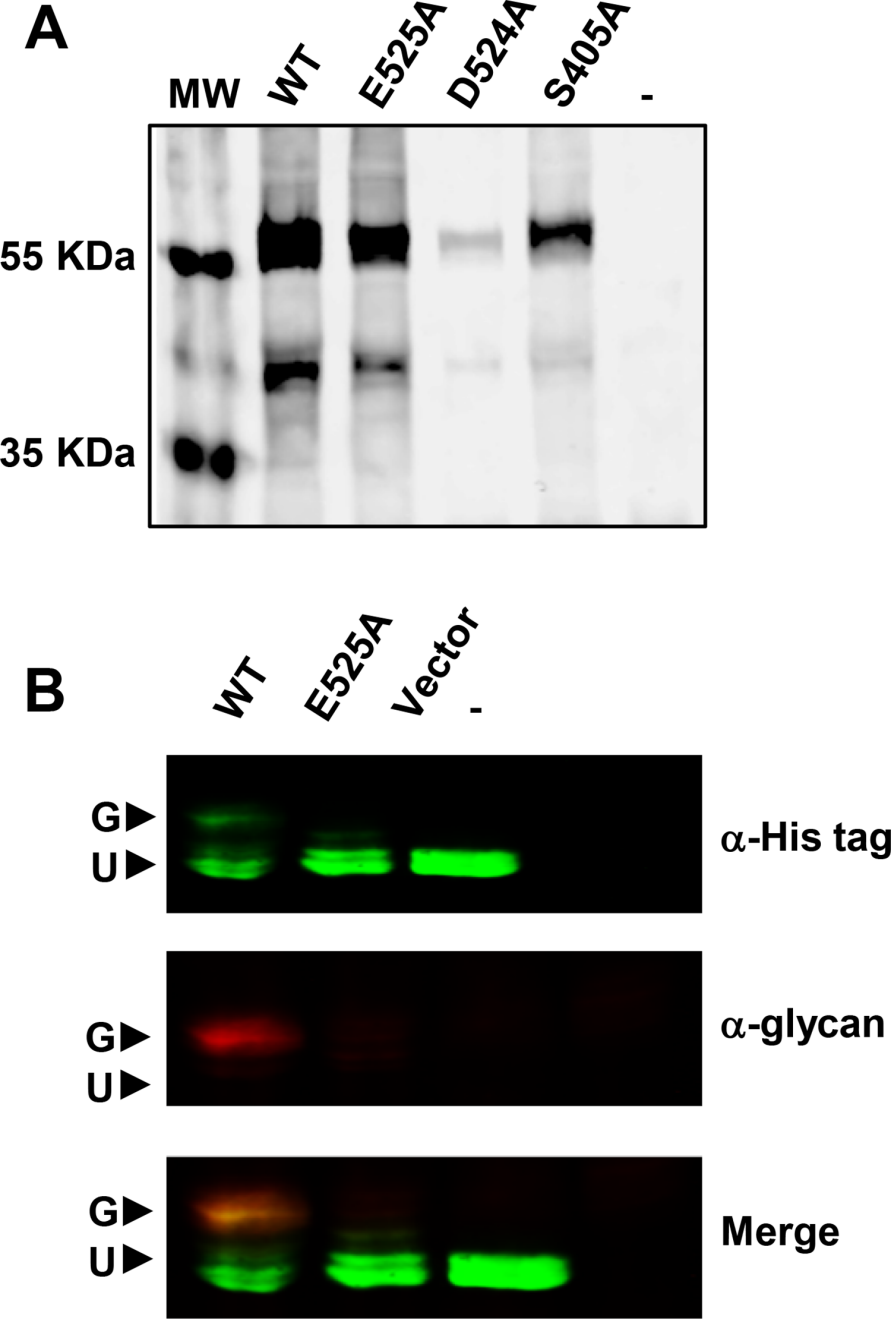
Generation of enzymatically inactive Wzk variant. (A) Expression of different *H*. *pylori* Wzk variants was induced by IPTG in *E*. *coli* DH5α for 4 h. Cell lysates from normalized cultures were separated on SDS-PAGE, followed by immunoblotting to detect the expression levels of Wzk mutants relative to wild type enzyme. Among the different Wzk variants, WzkE525A displayed expression levels comparable to that of wild type. (B) WzkE525A flippase activity is diminished *in vivo*. Both AcrA from *C*. *jejuni* and the accessory gene cluster required for its *N*-glycosylation were reconstituted in *E*. *coli*, with the exception of the native flippase. Flippase activity of Wzk and its E525A variant was tested by monitoring the glycosylation levels of *C*. *jejuni* AcrA via immunoblotting. Monoclonal anti-histidine was used to detect the expression of histidine-tagged AcrA (green), while the *C*. *jejuni* glycan was detected by the rabbit polyclonal anti-*C*. *jejuni* glycan (red). The glycosylated form of AcrA (G) is marked by the colocalization of both signals (yellow). Unglycosylated form of AcrA is marked U.

The ATPase components of ABC transporters contain highly conserved domains required for the binding and hydrolysis of ATP. Specifically, these ATPases contain Walker A domains with the consensus sequence GxxGxGK(T/S) and Walker B domains with the φφφφDE consensus, where x and φ represent any amino acid and a hydrophobic amino acid, respectively [60]. In Wzk (NCBI reference sequence WP_001074891), residues 398–405 (GHSGCGKS) and 520-525(ILVLDE) correspond to the expected Walker A and Walker B domains. Therefore, we expected that Wzk variants with changes in these motifs (S405A, D524A, or E525A) would be non-functional. Using site-directed mutagenesis, we constructed the respective mutant alleles of *wzk* in pIH23 and induced their expression in *E*. *coli* to analyze their stability. We found that while WzkE525A was produced to similar levels as wild-type Wzk, the WzkS405A and WzkD524A variants were produced at lower levels, suggesting that they have decreased stability or defects in folding (Fig 3A). Therefore, we proceeded in our analysis using the WzkE525A variant.

We tested the activity of WzkE525A in an *in vivo* flippase activity assay that takes advantage of the fact that Wzk is a flippase with relaxed substrate specificity. In this assay, the *E*. *coli* strain (SCM6) is used for the reconstitution of the *Campylobacter jejuni* protein *N-*glycosylation machinery [48]. AcrA, a well characterized *C*. *jejuni* glycoprotein, and the gene cluster responsible for its *N*-glycosylation, were expressed *in trans* in *E*. *coli* SCM6 with the exception of *C*. *jejuni* native flippase, PlgK, the ABC transporter that flips the lipid-linked N-oligosaccharide [31]. Because this strain lacks the endogenous flippases involved in O antigen and enterobacterial common antigens, AcrA is not glycosylated unless an active translocase is present. To test the flippase activity of WzkE525A, we introduced pIH23E525A and its control plasmids pEXT20 and pIH23 into strain SCM6, and monitored the glycosylation of AcrA in the three resulting strains. As previously reported [32], we observed that AcrA was glycosylated in SMC6 strain harboring pIH23 but not the vector control, demonstrating that wild-type Wzk is required to substitute for PglK (Fig 3B). Notably, we did not detect glycosylation of AcrA in strain SMC6 strain harboring pIH23E525A (Fig 3B), indicating that the E525A substitution abolished the flippase activity of Wzk, as would be expected from its inability to hydrolyze ATP.

Next, we tested for the ability of WzkE525A to substitute for MurJ in *E*. *coli* as described above. Introducing pIH23E525A into the Δ*murJ* (pRC7KanMurJ) reporter strain did not complement the Δ*murJ* mutation as indicated by the detection of only blue colonies of strain NR3649 in our MurJ complementation assay. Moreover, addition of various amounts of IPTG never promoted the generation of white (i.e. Δ*murJ*) colonies, indicating that WzkE525A cannot substitute for MurJ in *E*. *coli* (Fig 2A). We also tested the WzkS405A and WzkD524A variants in the event that they retained activity despite their folding defects but they did not complement the loss of MurJ in strain NR3649 and NR3648, respectively. Together, all of these results indicate that the ATPase activity of Wzk is required for its ability to function as a flippase of the Und-PP-linked precursor used in *N*-glycosylation, and to substitute for MurJ in *E*. *coli*. These findings support a model where Wzk complements the loss of MurJ by flipping lipid II in an ATP-dependent manner.

## Conclusions

Translocation of lipid II across the cytoplasmic membrane is an essential step in PG biogenesis [12]. The controversy surrounding the identification of lipid II flippases has hampered progress in understanding the mechanism of lipid II translocation [15]. Specifically, the debate surrounding MurJ was fueled by the fact that even though MurJ is essential for PG biogenesis in *E*. *coli* and other species, it is not in *B*. *subtilis* and *H*. *pylori* [29, 30, 41]. Here, we have demonstrated that like *B*. *subtilis*, *H*. *pylori* has another protein that is functionally redundant with MurJ [18]. In *H*. *pylori*, this protein is Wzk, a promiscuous Und-PP-oligosaccharide flippase [32]. Although MurJ flippase activity has not been reconstituted *in vitro*, the fact that Wzk is an ABC transporter that is functionally redundant with MurJ, together with previous findings on MurJ structure and function, strongly support that MurJ itself flips lipid II [20, 22, 23].

In addition, it is important to note AmJ and Wzk, which are functionally redundant with MurJ, show no evolutionary relationship with each other or MurJ [18]. These results have several implications. First, they demonstrate that cell viability should not be used as the only marker for the identification of flippases in gene clusters encoding essential glycans like PG. This type of argument has been used not only against MurJ [29, 30] but also against Rft1, a member of the MOP exporter superfamily that has been proposed and refuted to be a flippase required for N-glycosylation in eukaryotes [21, 61, 62]. Second, they highlight the need for studies that focus on understanding substrate specificity and mechanism of function of flippases of polyisoprenoid-linked oligosaccharides. Third, PG biogenesis has been the target of antibiotics that have been effective in the clinic [4]. In principle, MurJ has the potential to be the target of novel antibiotics and, indeed, it has been shown that small molecules that rapidly inactivate engineered MurJ variants cause cell lysis in *E*. *coli* [20]. However, the fact that some bacterial species encode factors that are functionally redundant with MurJ suggests that small molecule inhibitors of MurJ might have a narrower spectrum than anticipated. Therefore, this study highlights the importance of broadening the screening strategies for MurJ inhibitors to target other functionally-redundant proteins as well. Furthermore we suggest that Wzk is more promiscuous than other flippases such as Wzx. Wzx flippases are known to only flip lipid-linked oligosaccharides and the first sugar is important for recognition or activity [63–67]. However, Wzk appears to be able to flip long Lewis antigen chains in *Helicobacter pylori* and also shorter oligosaccharides, such as O antigens subunits or the N-heptasaccharide from *C*. *jejuni* [32], and, as we suggest here, lipid II, which is composed of sugars and amino acids. Recently, the three dimensional structure of PglK, the *C*. *jejuni* ortholog of Wzk, has been reported [40]. A flipping mechanism has been proposed in which the pyrophosphate moiety and the oligosaccharide are shielded from the lipid bilayer in a translocation cavity. Further work is required to understand how Wzk is able to shield both long polysaccharide chains and short oligosaccharide units.

## Supporting Information

S1 Fig Growth of *E*. *coli* strain NR3647 is dependent on expression of *wzk*. Growth of *E*. *coli* strain NR3647 [MG1655 Δ*lacIZYA*::*FRT* Δ*murJ*::*kan* (pIH23)] in LB broth supplemented with 40 μM IPTG (black-filled circles) or not (white-filled circles) as determined by OD_600_. The overall curve is representative of at least three independent experiments. A culture of NR3647 grown overnight in LB containing IPTG was diluted to OD_600_ 0.1 in LB either containing or not IPTG. Growth of NR3647 was dependent on the presence of IPTG. As Wzk was depleted in the absence of IPTG, OD_600_ stopped increasing. Complete lysis of the culture was not observed likely because of leaky expression of *wzk*.
(PDF)

S1 Table Strains used in this study. (PDF)

S2 Table Primers used in this study. (PDF)

## References

[1] Silhavy TJ, Kahne D, Walker S. The bacterial cell envelope. Cold Spring Harb Perspect Biol. 2010;2(5):a000414 10.1101/cshperspect.a000414 20452953 PMC2857177

[2] Typas A, Banzhaf M, Gross CA, Vollmer W. From the regulation of peptidoglycan synthesis to bacterial growth and morphology. Nat Rev Microbiol. 2012;10(2):123–36. 10.1038/nrmicro2677 . 22203377 PMC5433867

[3] Vollmer W, Blanot D, de Pedro MA. Peptidoglycan structure and architecture. FEMS Microbiol Rev. 2008;32(2):149–67. 10.1111/j.1574-6976.2007.00094.x . 18194336

[4] Bugg TD, Braddick D, Dowson CG, Roper DI. Bacterial cell wall assembly: still an attractive antibacterial target. Trends Biotechnol. 2011;29(4):167–73. Epub 2011/01/15. S0167-7799(10)00217-9 [pii] 10.1016/j.tibtech.2010.12.006 . 21232809

[5] Turner RD, Vollmer W, Foster SJ. Different walls for rods and balls: the diversity of peptidoglycan. Mol Microbiol. 2014;91(5):862–74. 10.1111/mmi.12513 24405365 PMC4015370

[6] Vollmer W, Seligman SJ. Architecture of peptidoglycan: more data and more models. Trends Microbiol. 2010;18(2):59–66. 10.1016/j.tim.2009.12.004 . 20060721

[7] Vollmer W, Bertsche U. Murein (peptidoglycan) structure, architecture and biosynthesis in *Escherichia coli*. Biochim Biophys Acta. 2008;1778(9):1714–34. 10.1016/j.bbamem.2007.06.007 . 17658458

[8] Chatterjee AN, Park JT. Biosynthesis of cell wall mucopeptide by a particulate fraction from *Staphylococcus aureus*. Proc Natl Acad Sci U S A. 1964;51:9–16. Epub 1964/01/01. 14106674 10.1073/pnas.51.1.9 PMC300596

[9] Meadow PM, Anderson JS, Strominger JL. Enzymatic polymerization of UDP-acetylmuramyl.L-ala.D-glu.L-lys.D-ala.D-ala and UDP-acetylglucosamine by a particulate enzyme from *Staphylococcus aureus* and its inhibition by antibiotics. Biochem Biophys Res Commun. 1964;14:382–7. Epub 1964/01/01. . 5836529 10.1016/s0006-291x(64)80014-0

[10] Mengin-Lecreulx D, Texier L, Rousseau M, van Heijenoort J. The murG gene of Escherichia coli codes for the UDP-N-acetylglucosamine: N-acetylmuramyl-(pentapeptide) pyrophosphoryl-undecaprenol N-acetylglucosamine transferase involved in the membrane steps of peptidoglycan synthesis. J Bacteriol. 1991;173(15):4625–36. 1649817 10.1128/jb.173.15.4625-4636.1991 PMC208138

[11] Barreteau H, Kovac A, Boniface A, Sova M, Gobec S, Blanot D. Cytoplasmic steps of peptidoglycan biosynthesis. FEMS Microbiol Rev. 2008;32(2):168–207. Epub 2008/02/13. FMR104 [pii] 10.1111/j.1574-6976.2008.00104.x . 18266853

[12] Bouhss A, Trunkfield AE, Bugg TD, Mengin-Lecreulx D. The biosynthesis of peptidoglycan lipid-linked intermediates. FEMS Microbiol Rev. 2008;32(2):208–33. . 18081839 10.1111/j.1574-6976.2007.00089.x

[13] Typas A, Banzhaf M, van den Berg van Saparoea B, Verheul J, Biboy J, Nichols RJ, et al Regulation of peptidoglycan synthesis by outer-membrane proteins. Cell. 2010;143(7):1097–109. Epub 2010/12/25. S0092-8674(10)01360-7 [pii] 10.1016/j.cell.2010.11.038 . 21183073 PMC3060616

[14] Young KD. Microbiology. A flipping cell wall ferry. Science. 2014;345(6193):139–40. 10.1126/science.1256585 . 25013047

[15] Ruiz N. Lipid Flippases for Bacterial Peptidoglycan Biosynthesis. Lipid Insights. 2015;8(Suppl 1):21–31. 10.4137/LPI.S31783 26792999 PMC4714577

[16] Inoue A, Murata Y, Takahashi H, Tsuji N, Fujisaki S, Kato J. Involvement of an essential gene, *mviN*, in murein synthesis in *Escherichia coli*. J Bacteriol. 2008;190(21):7298–301. Epub 2008/08/19. JB.00551-08 [pii] 10.1128/JB.00551-08 18708495 PMC2580715

[17] Ruiz N. Bioinformatics identification of MurJ (MviN) as the peptidoglycan lipid II flippase in *Escherichia coli*. Proc Natl Acad Sci U S A. 2008;105(40):15553–7. 10.1073/pnas.0808352105 18832143 PMC2563115

[18] Meeske AJ, Sham LT, Kimsey H, Koo BM, Gross CA, Bernhardt TG, et al MurJ and a novel lipid II flippase are required for cell wall biogenesis in *Bacillus subtilis*. Proc Natl Acad Sci U S A. 2015;112(20):6437–42. 10.1073/pnas.1504967112 25918422 PMC4443310

[19] Mohammadi T, van Dam V, Sijbrandi R, Vernet T, Zapun A, Bouhss A, et al Identification of FtsW as a transporter of lipid-linked cell wall precursors across the membrane. EMBO J. 2011;30(8):1425–32. 10.1038/emboj.2011.61 21386816 PMC3102273

[20] Sham LT, Butler EK, Lebar MD, Kahne D, Bernhardt TG, Ruiz N. Bacterial cell wall. MurJ is the flippase of lipid-linked precursors for peptidoglycan biogenesis. Science. 2014;345(6193):220–2. 10.1126/science.1254522 25013077 PMC4163187

[21] Hvorup RN, Winnen B, Chang AB, Jiang Y, Zhou XF, Saier MH Jr. The multidrug/oligosaccharidyl-lipid/polysaccharide (MOP) exporter superfamily. Eur J Biochem. 2003;270(5):799–813. . 12603313 10.1046/j.1432-1033.2003.03418.x

[22] Butler EK, Davis RM, Bari V, Nicholson PA, Ruiz N. Structure-function analysis of MurJ reveals a solvent-exposed cavity containing residues essential for peptidoglycan biogenesis in *Escherichia coli*. J Bacteriol. 2013;195(20):4639–49. Epub 2013/08/13. 10.1128/JB.00731-13 23935042 PMC3807429

[23] Butler EK, Tan WB, Joseph H, Ruiz N. Charge requirements of lipid II flippase activity in *Escherichia coli*. J Bacteriol. 2014;196(23):4111–9. 10.1128/JB.02172-14 25225268 PMC4248868

[24] Mohamed YF, Valvano MA. A Burkholderia cenocepacia MurJ (MviN) homolog is essential for cell wall peptidoglycan synthesis and bacterial viability. Glycobiology. 2014;24(6):564–76. 10.1093/glycob/cwu025 24688094 PMC4001712

[25] Rudnick PA, Arcondeguy T, Kennedy CK, Kahn D. *glnD* and *mviN* are genes of an essential operon in *Sinorhizobium meliloti*. J Bacteriol. 2001;183(8):2682–5. Epub 2001/03/29. 10.1128/JB.183.8.2682-2685.2001 11274131 PMC95188

[26] Huber J, Donald RG, Lee SH, Jarantow LW, Salvatore MJ, Meng X, et al Chemical genetic identification of peptidoglycan inhibitors potentiating carbapenem activity against methicillin-resistant *Staphylococcus aureus*. Chem Biol. 2009;16(8):837–48. Epub 2009/09/01. S1074-5521(09)00241-5 [pii] 10.1016/j.chembiol.2009.05.012 . 19716474

[27] Thanassi JA, Hartman-Neumann SL, Dougherty TJ, Dougherty BA, Pucci MJ. Identification of 113 conserved essential genes using a high-throughput gene disruption system in *Streptococcus pneumoniae*. Nucleic Acids Res. 2002;30(14):3152–62. 12136097 10.1093/nar/gkf418 PMC135739

[28] Ruiz N. *Streptococcus pyogenes* YtgP (Spy_0390) complements *Escherichia coli* strains depleted of the putative peptidoglycan flippase MurJ. Antimicrob Agents Chemother. 2009;53(8):3604–5. 10.1128/AAC.00578-09 19528283 PMC2715597

[29] Fay A, Dworkin J. *Bacillus subtilis* homologs of MviN (MurJ), the putative *Escherichia coli* lipid II flippase, are not essential for growth. J Bacteriol. 2009;191(19):6020–8. Epub 2009/08/12. JB.00605-09 [pii] 10.1128/JB.00605-09 19666716 PMC2747889

[30] Vasudevan P, McElligott J, Attkisson C, Betteken M, Popham DL. Homologues of the *Bacillus subtilis* SpoVB protein are involved in cell wall metabolism. J Bacteriol. 2009;191(19):6012–9. Epub 2009/08/04. JB.00604-09 [pii] 10.1128/JB.00604-09 19648239 PMC2747891

[31] Alaimo C, Catrein I, Morf L, Marolda CL, Callewaert N, Valvano MA, et al Two distinct but interchangeable mechanisms for flipping of lipid-linked oligosaccharides. EMBO J. 2006;25(5):967–76. 10.1038/sj.emboj.7601024 16498400 PMC1409731

[32] Hug I, Couturier MR, Rooker MM, Taylor DE, Stein M, Feldman MF. *Helicobacter pylori* lipopolysaccharide is synthesized via a novel pathway with an evolutionary connection to protein N-glycosylation. PLoS Pathog. 2010;6(3):e1000819 10.1371/journal.ppat.1000819 20333251 PMC2841628

[33] Taylor VL, Huszczynski SM, Lam JS. Membrane Translocation and Assembly of Sugar Polymer Precursors. Curr Top Microbiol Immunol. 2016 10.1007/82_2015_5014 . 26853690

[34] Hug I, Feldman MF. Analogies and homologies in lipopolysaccharide and glycoprotein biosynthesis in bacteria. Glycobiology. 2011;21(2):138–51. Epub 2010/09/28. 10.1093/glycob/cwq148 . 20871101

[35] Islam ST, Lam JS. Wzx flippase-mediated membrane translocation of sugar polymer precursors in bacteria. Environ Microbiol. 2013;15(4):1001–15. 10.1111/j.1462-2920.2012.02890.x . 23016929

[36] Cuthbertson L, Kos V, Whitfield C. ABC transporters involved in export of cell surface glycoconjugates. Microbiol Mol Biol Rev. 2010;74(3):341–62. Epub 2010/09/02. 74/3/341 [pii] 10.1128/MMBR.00009-10 20805402 PMC2937517

[37] Yan A, Guan Z, Raetz CR. An undecaprenyl phosphate-aminoarabinose flippase required for polymyxin resistance in *Escherichia coli*. J Biol Chem. 2007;282(49):36077–89. 10.1074/jbc.M706172200 17928292 PMC2613183

[38] Sanabria-Valentin E, Colbert MT, Blaser MJ. Role of *futC* slipped strand mispairing in *Helicobacter pylori* Lewisy phase variation. Microbes Infect. 2007;9(14–15):1553–60. 10.1016/j.micinf.2007.08.011 18024122 PMC2245886

[39] Wang G, Ge Z, Rasko DA, Taylor DE. Lewis antigens in *Helicobacter pylori*: biosynthesis and phase variation. Mol Microbiol. 2000;36(6):1187–96. . 10931272 10.1046/j.1365-2958.2000.01934.x

[40] Perez C, Gerber S, Boilevin J, Bucher M, Darbre T, Aebi M, et al Structure and mechanism of an active lipid-linked oligosaccharide flippase. Nature. 2015;524(7566):433–8. 10.1038/nature14953 . 26266984

[41] Sycuro LK, Rule CS, Petersen TW, Wyckoff TJ, Sessler T, Nagarkar DB, et al Flow cytometry-based enrichment for cell shape mutants identifies multiple genes that influence *Helicobacter pylori* morphology. Mol Microbiol. 2013;90(4):869–83. 10.1111/mmi.12405 24112477 PMC3844677

[42] Silhavy TJ, Berman ML, Enquist LW. Experiments with gene fusions Cold Spring Harbor, NY: Cold Spring Harbor Laboratory; 1984.

[43] Yu D, Ellis HM, Lee EC, Jenkins NA, Copeland NG, Court DL. An efficient recombination system for chromosome engineering in *Escherichia coli*. Proc Natl Acad Sci U S A. 2000;97(11):5978–83. Epub 2000/05/17. 10.1073/pnas.100127597 10811905 PMC18544

[44] Horton RM. PCR-mediated recombination and mutagenesis. SOEing together tailor-made genes. Mol Biotechnol. 1995;3(2):93–9. 10.1007/BF02789105 . 7620981

[45] Menard R, Molinas C, Arthur M, Duval J, Courvalin P, Leclercq R. Overproduction of 3'-aminoglycoside phosphotransferase type I confers resistance to tobramycin in *Escherichia coli*. Antimicrob Agents Chemother. 1993;37(1):78–83. 8381641 10.1128/aac.37.1.78 PMC187608

[46] Humbert O, Salama NR. The Helicobacter pylori HpyAXII restriction-modification system limits exogenous DNA uptake by targeting GTAC sites but shows asymmetric conservation of the DNA methyltransferase and restriction endonuclease components. Nucleic Acids Res. 2008;36(21):6893–906. 10.1093/nar/gkn718 18978016 PMC2588503

[47] Salama NR, Shepherd B, Falkow S. Global transposon mutagenesis and essential gene analysis of *Helicobacter pylori*. J Bacteriol. 2004;186(23):7926–35. 10.1128/JB.186.23.7926-7935.2004 15547264 PMC529078

[48] Musumeci MA, Faridmoayer A, Watanabe Y, Feldman MF. Evaluating the role of conserved amino acids in bacterial O-oligosaccharyltransferases by in vivo, in vitro and limited proteolysis assays. Glycobiology. 2014;24(1):39–50. 10.1093/glycob/cwt087 . 24092836

[49] Liu D, Reeves PR. Escherichia coli K12 regains its O antigen. Microbiology. 1994;140 (Pt 1):49–57. 10.1099/13500872-140-1-49 . 7512872

[50] Linton D, Dorrell N, Hitchen PG, Amber S, Karlyshev AV, Morris HR, et al Functional analysis of the *Campylobacter jejuni* N-linked protein glycosylation pathway. Mol Microbiol. 2005;55(6):1695–703. 10.1111/j.1365-2958.2005.04519.x . 15752194

[51] Dykxhoorn DM, St Pierre R, Linn T. A set of compatible tac promoter expression vectors. Gene. 1996;177(1–2):133–6. . 8921858 10.1016/0378-1119(96)00289-2

[52] Datsenko KA, Wanner BL. One-step inactivation of chromosomal genes in *Escherichia coli* K-12 using PCR products. Proc Natl Acad Sci U S A. 2000;97(12):6640–5. Epub 2000/06/01. 10.1073/pnas.120163297 10829079 PMC18686

[53] Blattner FR, Plunkett G 3rd, Bloch CA, Perna NT, Burland V, Riley M, et al The complete genome sequence of *Escherichia coli* K-12. Science. 1997;277(5331):1453–62. . 9278503 10.1126/science.277.5331.1453

[54] Baba T, Ara T, Hasegawa M, Takai Y, Okumura Y, Baba M, et al Construction of *Escherichia coli* K-12 in-frame, single-gene knockout mutants: the Keio collection. Mol Syst Biol. 2006;2:2006.0008 . 16738554 10.1038/msb4100050 PMC1681482

[55] Cherepanov PP, Wackernagel W. Gene disruption in *Escherichia coli*: TcR and KmR cassettes with the option of Flp-catalyzed excision of the antibiotic-resistance determinant. Gene. 1995;158(1):9–14. . 7789817 10.1016/0378-1119(95)00193-a

[56] Altschul SF, Madden TL, Schaffer AA, Zhang J, Zhang Z, Miller W, et al Gapped BLAST and PSI-BLAST: a new generation of protein database search programs. Nucleic Acids Res. 1997;25(17):3389–402. 9254694 10.1093/nar/25.17.3389 PMC146917

[57] Jorgenson MA, Kannan S, Laubacher ME, Young KD. Dead-end intermediates in the enterobacterial common antigen pathway induce morphological defects in *Escherichia coli* by competing for undecaprenyl phosphate. Mol Microbiol. 2016;100(1):1–14. 10.1111/mmi.13284 26593043 PMC4845916

[58] Ranjit DK, Young KD. Colanic acid intermediates prevent *de novo* shape recovery of *Escherichia coli* spheroplasts, calling into question biological roles previously attributed to colanic acid. J Bacteriol. 2016;198(8):1230–40. 10.1128/JB.01034-15 . 26833417 PMC4859594

[59] de Boer PA, Crossley RE, Rothfield LI. A division inhibitor and a topological specificity factor coded for by the minicell locus determine proper placement of the division septum in *E*. *coli*. Cell. 1989;56(4):641–9. . 2645057 10.1016/0092-8674(89)90586-2

[60] Davidson AL, Dassa E, Orelle C, Chen J. Structure, function, and evolution of bacterial ATP-binding cassette systems. Microbiol Mol Biol Rev. 2008;72(2):317–64, table of contents. 10.1128/MMBR.00031-07 18535149 PMC2415747

[61] Helenius J, Ng DT, Marolda CL, Walter P, Valvano MA, Aebi M. Translocation of lipid-linked oligosaccharides across the ER membrane requires Rft1 protein. Nature. 2002;415(6870):447–50. Epub 2002/01/25. 10.1038/415447a 415447a [pii]. . 11807558

[62] Jelk J, Gao N, Serricchio M, Signorell A, Schmidt RS, Bangs JD, et al Glycoprotein biosynthesis in a eukaryote lacking the membrane protein Rft1. J Biol Chem. 2013;288(28):20616–23. 10.1074/jbc.M113.479642 23720757 PMC3711325

[63] Marolda CL, Vicarioli J, Valvano MA. Wzx proteins involved in biosynthesis of O antigen function in association with the first sugar of the O-specific lipopolysaccharide subunit. Microbiology. 2004;150(Pt 12):4095–105. 10.1099/mic.0.27456-0 . 15583162

[64] Islam ST, Lam JS. Synthesis of bacterial polysaccharides via the Wzx/Wzy-dependent pathway. Can J Microbiol. 2014;60(11):697–716. 10.1139/cjm-2014-0595 . 25358682

[65] Feldman MF, Marolda CL, Monteiro MA, Perry MB, Parodi AJ, Valvano MA. The activity of a putative polyisoprenol-linked sugar translocase (Wzx) involved in *Escherichia coli* O antigen assembly is independent of the chemical structure of the O repeat. J Biol Chem. 1999;274(49):35129–38. . 10574995 10.1074/jbc.274.49.35129

[66] Hong Y, Reeves PR. Diversity of o-antigen repeat unit structures can account for the substantial sequence variation of wzx translocases. J Bacteriol. 2014;196(9):1713–22. 10.1128/JB.01323-13 24532778 PMC3993327

[67] Hong Y, Cunneen MM, Reeves PR. The Wzx translocases for Salmonella enterica O-antigen processing have unexpected serotype specificity. Mol Microbiol. 2012;84(4):620–30. 10.1111/j.1365-2958.2012.08048.x . 22497246

