# Correction: The O-Antigen Flippase Wzk Can Substitute for MurJ in Peptidoglycan Synthesis in *Helicobacter pylori* and *Escherichia coli*

**DOI:** 10.1371/journal.pone.0170518

**Published:** 2017-01-23

**Authors:** Wael Elhenawy, Rebecca M. Davis, Jutta Fero, Nina R. Salama, Mario F. Feldman, Natividad Ruiz

The fifth authors name is spelled incorrectly. The correct name is Mario F. Feldman. The correct citation is: Elhenawy W, Davis RM, Fero J, Salama NR, Feldman MF, Ruiz N (2016) The O-Antigen Flippase Wzk Can Substitute for MurJ in Peptidoglycan Synthesis in *Helicobacter pylori* and *Escherichia coli*. PLoS ONE 11(8): e0161587. doi:10.1371/journal.pone.0161587.

## References

[1] Elhenawy W, Davis RM, Fero J, Salama NR, Felman MF, Ruiz N (2016) The O-Antigen Flippase Wzk Can Substitute for MurJ in Peptidoglycan Synthesis in *Helicobacter pylori* and *Escherichia coli*. PLoS ONE 11(8): e0161587 doi: 10.1371/journal.pone.0161587 27537185 10.1371/journal.pone.0161587 PMC4990322

