# Supplementary figures and images for: The O-Antigen Flippase Wzk Can Substitute for MurJ in Peptidoglycan Synthesis in Helicobacter pylori and Escherichia coli
Source: PLoS One. 2016 Aug 18;11(8):e0161587. doi: 10.1371/journal.pone.0161587 (PMC4990322; doi:10.1371/journal.pone.0161587)

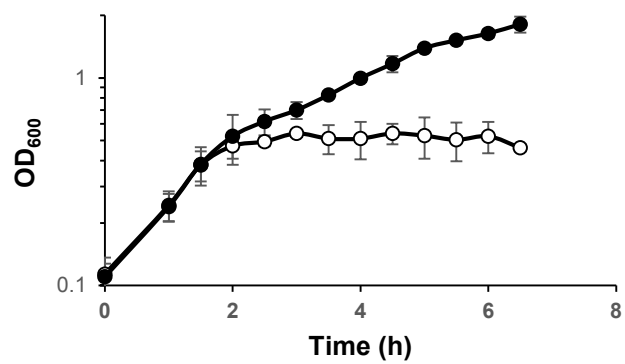

**S1 Fig.**

Supplement: S1 Fig — Growth of E. coli strain NR3647 [MG1655 ΔlacIZYA::FRT ΔmurJ::kan (pIH23)] in LB broth supplemented with 40 μM IPTG (black-filled circles) or not (white-filled circles) as determined by OD600. The overall curve is representative of at least three independent experiments. A culture of NR3647 grown overnight in LB containing IPTG was diluted to OD600 0.1 in LB either containing or not IPTG. Growth of NR3647 was dependent on the presence of IPTG. As Wzk was depleted in the absence of IPTG, OD600 stopped increasing. Complete lysis of the culture was not observed likely because of leaky expression of wzk. (PDF) [file pone.0161587.s001.pdf]
