# Supplementary material for: The O-Antigen Flippase Wzk Can Substitute for MurJ in Peptidoglycan Synthesis in Helicobacter pylori and Escherichia coli
Source: PLoS One. 2016 Aug 18;11(8):e0161587. doi: 10.1371/journal.pone.0161587 (PMC4990322; doi:10.1371/journal.pone.0161587)
Supplement: S1 Table — (PDF) [file pone.0161587.s002.pdf]

**S1 Table. Strains used in this study**

| Strain name | Relevant genotype | Reference |
|---|---|---|
| LSH100 | Wild-type *H. pylori*, NSH57 with G27 *fliM* allele | [1] |
| TSH1 | LSH100 *murJ::cat* | [2] |
| TSH13 | LSH100 *murJ::cat rdxA:murJ* | [2] |
| NSH203 | LSH100 *wzk::aphA3* | This work |
| NSH207 | LSH100 *wacA::cat* | This work |
| NSH208 | LSH100 *wecA::cat wzk::aphA3* | This work |
| SCM6 | SΦ874 Δ*waaL*, Δ*wecA* | [3] |
| MG1655 | Wild-type *E. coli* K-12 *rph1 ilvG rfb-50* | [4] |
| TB28 | MG1655 Δ*lacIYZA*::*FRT* | [5] |
| NR754 | Δ (*lac*)U169, *strA*, *thi* | [6] |
| NR1648 | NR754 Δ*murJ*::*kan* (pRC7MurJ) | [7] |
| NR2865 | MG1655 Δ*proC* | This work |
| NR2869 | MG1655 Δ*lacIYZA*::*FRT* | This work |
| NR2874 | NR2869 Δ*pyrC*::*FRT* (pRC7KanMurJ) | This work |
| NR2890 | MG1655 Δ*lacIYZA*::*FRT* Δ*murJ*::*kan* (pRC7KanMurJ) | This work |
| NR2919 | NR2890 (pEXT20) | This work |
| NR2920 | NR2890 (pIH23) | This work |
| NR3647 | MG1655 Δ*lacIZYA*::*FRT* Δ*murJ*::*kan* (pIH23) | This work |
| NR3648 | NR2890 (pIH23D524A) | This work |
| NR3649 | NR2890 (pIH23S405A) | This work |
| NR3650 | NR2890 (pIH23E525A) | This work |
| DH5α | *fhuA2 lac(del)U169 phoA glnV44 Φ80' lacZ(del)M15 gyrA96 recA1 relA1 endA1 thi-1 hsdR17* | [8] |
| DY378 | Recombineering strain; λcI857 Δ(*cro-bioA*) | [9] |

# References


1. Lowenthal AC, Hill M, Sycuro LK, Mehmood K, Salama NR, Ottemann KM. Functional analysis of the *Helicobacter pylori* flagellar switch proteins. J Bacteriol. 2009;191(23):7147-56. doi: 10.1128/JB.00749-09. PubMed PMID: 19767432; PubMed Central PMCID: PMCPMC2786559.

2. Sycuro LK, Rule CS, Petersen TW, Wyckoff TJ, Sessler T, Nagarkar DB, et al. Flow cytometry-based enrichment for cell shape mutants identifies multiple genes that influence *Helicobacter pylori* morphology. Mol Microbiol. 2013;90(4):869-83. doi: 10.1111/mmi.12405. PubMed PMID: 24112477; PubMed Central PMCID: PMCPMC3844677.

3. Musumeci MA, Faridmoayer A, Watanabe Y, Feldman MF. Evaluating the role of conserved amino acids in bacterial O-oligosaccharyltransferases by *in vivo*, in vitro and limited proteolysis assays. Glycobiology. 2014;24(1):39-50. doi: 10.1093/glycob/cwt087. PubMed PMID: 24092836.

4. Blattner FR, Plunkett G, 3rd, Bloch CA, Perna NT, Burland V, Riley M, et al. The complete genome sequence of *Escherichia coli* K-12. Science. 1997;277(5331):1453-62. PubMed PMID: 9278503.

5. Bernhardt TG, de Boer PA. The Escherichia coli amidase AmiC is a periplasmic septal ring component exported via the twin-arginine transport pathway. Mol Microbiol. 2003;48(5):1171-82. Epub 2003/06/06. PubMed PMID: 12787347.

6. Ruiz N, Gronenberg LS, Kahne D, Silhavy TJ. Identification of two inner-membrane proteins required for the transport of lipopolysaccharide to the outer membrane of *Escherichia coli*. Proc Natl Acad Sci U S A. 2008;105(14):5537-42. Epub 2008/04/01. doi: 0801196105 [pii]10.1073/pnas.0801196105. PubMed PMID: 18375759; PubMed Central PMCID: PMC2291135.

7. Butler EK, Davis RM, Bari V, Nicholson PA, Ruiz N. Structure-function analysis of MurJ reveals a solvent-exposed cavity containing residues essential for peptidoglycan biogenesis in *Escherichia coli*. J Bacteriol. 2013;195(20):4639-49. Epub 2013/08/13. doi: 10.1128/JB.00731-13. PubMed PMID: 23935042; PubMed Central PMCID: PMC3807429.

8. Grant SG, Jessee J, Bloom FR, Hanahan D. Differential plasmid rescue from transgenic mouse DNAs into *Escherichia coli* methylation-restriction mutants. Proc Natl Acad Sci U S A. 1990;87(12):4645-9. PubMed PMID: 2162051; PubMed Central PMCID: PMCPMC54173.

9. Yu D, Ellis HM, Lee EC, Jenkins NA, Copeland NG, Court DL. An efficient recombination system for chromosome engineering in *Escherichia coli*. Proc Natl Acad Sci U S A. 2000;97(11):5978-83. Epub 2000/05/17. doi: 10.1073/pnas.100127597. PubMed PMID: 10811905; PubMed Central PMCID: PMC18544.
