# Supplementary material for: The O-Antigen Flippase Wzk Can Substitute for MurJ in Peptidoglycan Synthesis in Helicobacter pylori and Escherichia coli
Source: PLoS One. 2016 Aug 18;11(8):e0161587. doi: 10.1371/journal.pone.0161587 (PMC4990322; doi:10.1371/journal.pone.0161587)
Supplement: S2 Table — (PDF) [file pone.0161587.s003.pdf]

S2 Table. Primers used in this study

Primer name	Sequence (5' to 3')
1153_for_out	GATTCTCGCCTTACAAAT
1153#2a	GAGCCATTTTGCACACC
1153Kan#3	TTCATAGAGTAATTCTGTGCAAAGTGGTGTAAATGA
1153Kan#4	TTGTTTTAGTACCTAGATACGCTAGCGGATATTATT
1516_rev_in	GGTTTCTATGCAGGTTTT
1519_rev_in	AGACACTTCAATGCCTTT
WzkS405AFw1	GCCATAGCGGGTGCGGAAAAGCGACGCTGGCGGATATTATTATG
WzkS405ARv1	CATAATAATATCCGCCAGCGTCGCTTTTCCGCACCCGCTATGGC
WzkD524AFw1	CTGAAATTTTGGTTTTAGCGGAAGCCACTTCAGCCCTAG
WzkD524ARv1	CTAGGGCTGAAGTGGCTTCCGCTAAAACCAAAATTTCAG
WzkE525AFw1	GAAATTTTGGTTTTAGATGCGGCCACTTCAGCCCTAGAC
WzkE525ARv1	GTCTAGGGCTGAAGTGGCCGCATCTAAAACCAAAATTTC
BlaP1	ATGAGTATTCAACATTTCCGTGTCGCCCTTATTCCCTTTTTTGCGG CATTTTGCCTTCCTGTTTTTGCTCGTGTAGGCTGGAGCTGCTTC
BlaP2	TTACCAATGCTTAATCAGTGAGGCACCTATCTCAGCGATCTGTCT ATTTCGTTCATCCATAGTTGCCTGACATATGAATATCCTCCTTA
